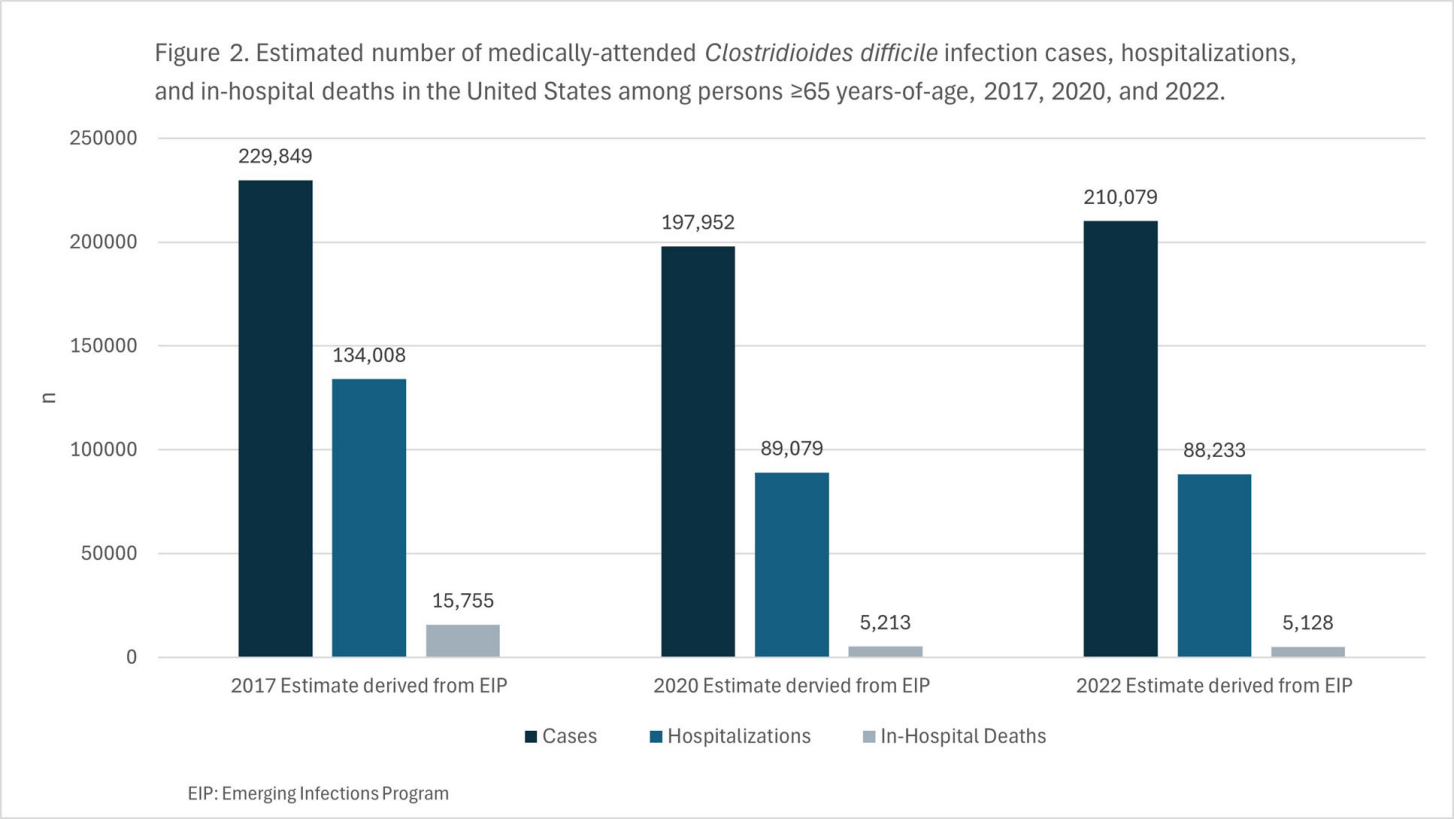# 123 Improving Blood Culture Pathogen Detection, Reducing Contamination, and Timeliness in the Emergency Department

**DOI:** 10.1017/ash.2026.10537

**Published:** 2026-06-23

**Authors:** Natalie McCarthy, Frederick Angulo, Stephanie Duench, Steven Shen, Constantina Boikos, Jamie Findlow

**Affiliations:** 1 Pfizer, Vaccines RWE Epidemiology; 2 Pfizer Vaccines; 3 Pfizer

## Abstract

**Background:** Clostridioides difficile infection (CDI) is an important cause of morbidity and mortality worldwide, and in the United States (US) has been declared an “urgent public health threat” by the Centers for Disease Control and Prevention (CDC). The most recently published CDC estimate of the number of CDI infections, hospitalizations, and in-hospital deaths in the US is for 2017. However, CDC’s Emerging Infections Program (EIP) provides ongoing surveillance data; we used EIP annual reports to derive estimates of the number of medically-attended CDI cases, hospitalizations, and deaths in the US in 2017-2022. **Methods:** Age- and healthcare-associated and community-associated stratified, population-based incidence rates of laboratory-confirmed CDI cases from EIP annual reports were multiplied by age-stratified US Census estimates to derive national estimates of CDI cases in 2017-2022. The percentage of CDI cases hospitalized on the day of or within 6 days of specimen collection, and the in-hospital mortality rates reported in EIP annual reports, were multiplied by the estimated number of CDI cases to calculate hospitalizations and in-hospital deaths for the same years. A similar approach was used to estimate cases, hospitalizations, and in-hospital deaths among persons ≥65 years-of-age. All estimates were compared with published 2017 CDC estimates. **Results:** We estimated that there were 449,946 laboratory-confirmed CDI cases in 2017, compared to 462,100 cases in the CDC estimate; estimated hospitalizations and in-hospital deaths in 2017 were also similar to the CDC estimate (Figure 1). We estimated 406,130 CDI cases in 2022; 210,079 CDI cases in persons ≥65 years-of-age. We also estimated 170,575 CDI hospitalizations and 9,914 in-hospital deaths in 2022; 88,233 hospitalizations and 5,128 in-hospital deaths in persons ≥65 years-of-age. From 2017-2020, the estimated number of CDI cases, hospitalizations, and in-hospital deaths declined by 22%, 33%, and 58%, respectively, and from 2020-2022 estimates increased by 16%, 8%, and 8%, respectively. Among persons ≥65 years-of-age, estimated CDI cases declined by 14% from 2017-2020, and increased by 6% from 2020-2022; from 2017-2020, estimated hospitalizations and in-hospital deaths declined by 34% and 67%, respectively, and remained stable from 2020-2022 (Figure 2). **Conclusion:** CDI burden estimates from EIP annual reports were comparable to CDC estimates, supporting the use of EIP data to derive national estimates. The US CDI disease burden is high and has increased since the COVID-19 pandemic. Interventions are needed to reduce the US CDI disease burden, particularly in older age groups.

**Figure:**
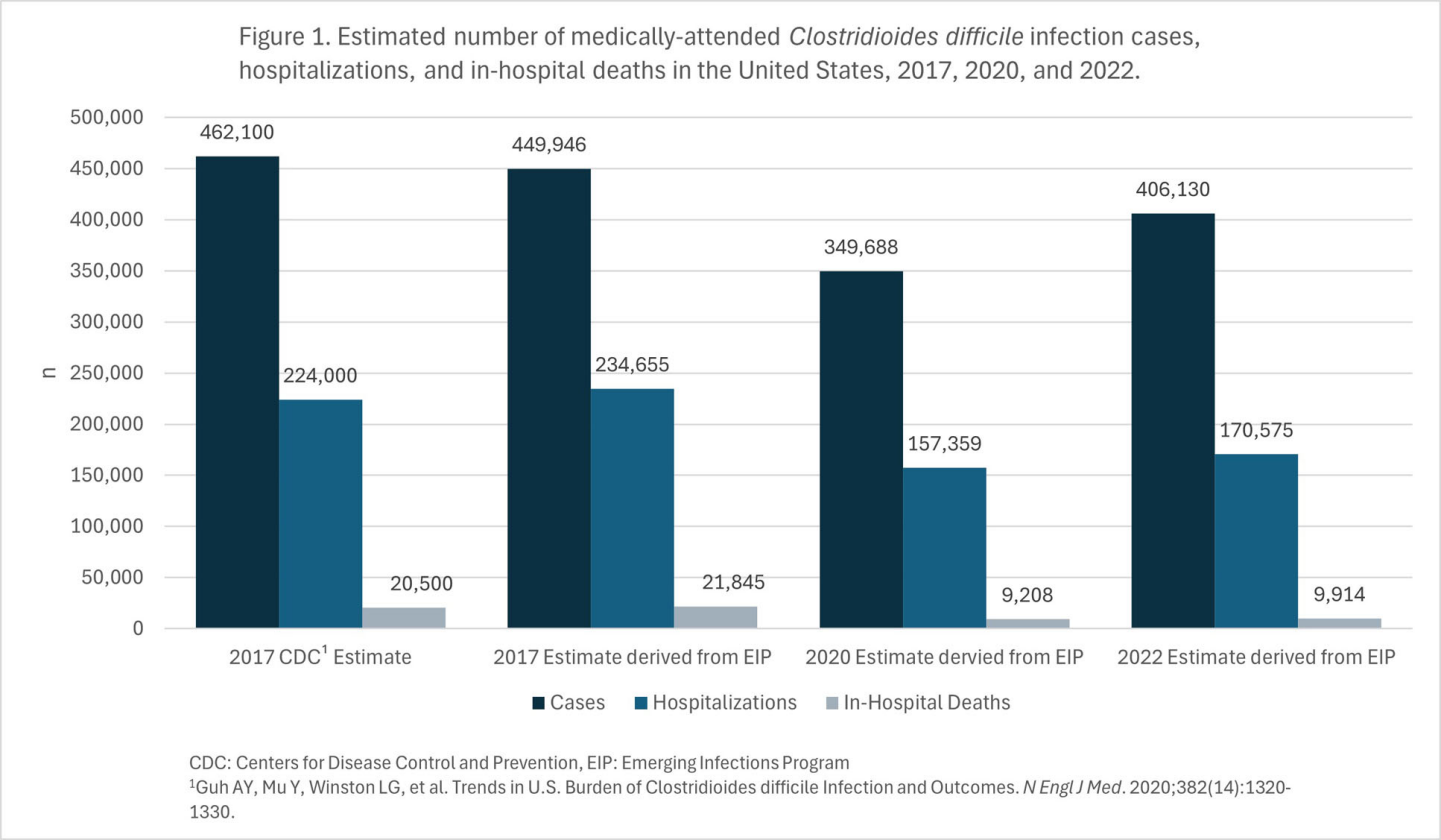

**Figure:**